# Gonadotropin-releasing hormone type II antagonist induces apoptosis in MCF-7 and triple-negative MDA-MB-231 human breast cancer cells *in vitro *and *in vivo*

**DOI:** 10.1186/bcr2606

**Published:** 2010-07-14

**Authors:** Carsten Gründker, Crispin Föst, Stefanie Fister, Nadine Nolte, Andreas R Günthert, Günter Emons

**Affiliations:** 1 Department of Gynecology and Obstetrics, Georg-August-University, Robert-Koch-Street 40, 37075 Göttingen, Germany

## Abstract

**Introduction:**

Triple-negative breast cancer does not express estrogen and progesterone receptors, and no overexpression/amplification of the *HER2-neu *gene occurs. Therefore, this subtype of breast cancer lacks the benefits of specific therapies that target these receptors. Today chemotherapy is the only systematic therapy for patients with triple-negative breast cancer. About 50% to 64% of human breast cancers express receptors for gonadotropin-releasing hormone (GnRH), which might be used as a target. New targeted therapies are warranted. Recently, we showed that antagonists of gonadotropin-releasing hormone type II (GnRH-II) induce apoptosis in human endometrial and ovarian cancer cells *in vitro *and *in vivo*. This was mediated through activation of stress-induced mitogen-activated protein kinases (MAPKs) p38 and c-Jun N-terminal kinase (JNK), followed by activation of proapoptotic protein Bax, loss of mitochondrial membrane potential, and activation of caspase-3. In the present study, we analyzed whether GnRH-II antagonists induce apoptosis in MCF-7 and triple-negative MDA-MB-231 human breast cancer cells that express GnRH receptors. In addition, we ascertained whether knockdown of GnRH-I receptor expression affects GnRH-II antagonist-induced apoptosis and apoptotic signaling.

**Methods:**

Induction of apoptosis was analyzed by measurement of the loss of mitochondrial membrane potential. Apoptotic signaling was measured with quantification of activated MAPK p38 and caspase-3 by using the Western blot technique. GnRH-I receptor protein expression was inhibited by using the antisense knockdown technique. *In vivo *experiments were performed by using nude mice bearing xenografted human breast tumors.

**Results:**

We showed that treatment of MCF-7 and triple-negative MDA-MB-231 human breast cancer cells with a GnRH-II antagonist results in apoptotic cell death *in vitro *via activation of stress-activated MAPK p38 and loss of mitochondrial membrane potential. In addition, we showed GnRH-II antagonist-induced activation of caspase-3 in MDA-MB-231 human breast cancer cells. After knockdown of GnRH-I receptor expression, GnRH-II antagonist-induced apoptosis and apoptotic signaling was only slightly reduced, indicating that an additional pathway mediating the effects of GnRH-II antagonists may exist. The GnRH-I receptor seems not to be the only target of GnRH-II antagonists. The antitumor effects of the GnRH-II antagonist could be confirmed in nude mice. The GnRH-II antagonist inhibited the growth of xenotransplants of human breast cancers in nude mice completely, without any apparent side effects.

**Conclusions:**

GnRH-II antagonists seem to be suitable drugs for an efficacious and less-toxic endocrine therapy for breast cancers, including triple-negative breast cancers.

## Introduction

Breast cancer is the most frequent malignant disease in women, with more than 1,000,000 new cases and 370,000 deaths yearly worldwide [1]. About 75$ to 80% of breast cancers are hormone-receptor positive and express estrogen and progesterone receptors [2,3]. Approximately 15% to 20% of breast cancers overexpress/amplify the *HER2-neu *gene, with about half of these co-expressing steroid-hormone receptors. For patients with hormone-receptor-positive or *HER2-neu*-positive tumors, effective targeted therapies have been developed. About 10% to 15% of breast cancers do not express either estrogen and progesterone receptor and also do not overexpress/amplify the *HER2-neu *gene [4-6]. These so-called triple-negative breast cancers lack the benefits of specific therapies that target these receptors. Triple-negative breast cancer is relatively chemosensitive to conventional cytotoxic agents such as cisplatin, but the effectiveness is for only a short duration. Therefore, the development of new therapies is of great interest.

The expression of gonadotropin-releasing hormone (GnRH-I) and its receptor as a part of a negative autocrine/paracrine regulatory mechanism of cell proliferation has been demonstrated in a number of malignant tumors, including cancers of endometrium, ovary, and breast [7]. In these cancers, the *in vitro *proliferation can be inhibited by agonistic analogues of GnRH-I in a dose- and time-dependent manner [7-11]. GnRH-I antagonists also have marked antiproliferative activity in most endometrial, ovarian, and breast cancer cell lines tested *in vitro*, indicating that the dichotomy of GnRH agonists and antagonists might not apply to the GnRH system in cancer cells [7-11].

Besides GnRH-I, a second structural variant of GnRH exists in mammals. GnRH-II is totally conserved in structure from fish to mammals. It differs from GnRH-I in three amino acids. GnRH-II receptor was found in different species, including nonhuman primates. Its existence in the human is controversial. Several lines of evidence, however, exist for a functional GnRH-II receptor [12]. GnRH-II has antiproliferative effects on human endometrial, ovarian, and breast cancer cells that are significantly greater than those of the superactive GnRH-I agonist triptorelin [13]. Induction of apoptosis is not involved in the downregulation of cancer cell proliferation induced by agonists of GnRH-I or GnRH-II [7]. GnRH-I and GnRH-II agonists rather inhibit mitogenic signal transduction of growth-factor receptors via activation of a phosphotyrosine phosphatase, resulting in downregulation of cancer cell proliferation [14-16].

Recently, we showed that antagonistic analogues of GnRH-II induced apoptotic cell death in human endometrial and ovarian cancer cells *in vitro*, via dose-dependent loss of mitochondrial membrane potential and activation of caspase-3 [17]. These antitumor effects could be confirmed in nude mice. GnRH-II antagonists significantly inhibited the growth of xenotransplants of human endometrial and ovarian cancers in nude mice, without any apparent side effects [17]. Apoptosis induced by GnRH-II antagonists is mediated through the intrinsic apoptotic pathway via stress-induced MAPKs p38- and JNK-induced activation of the pro-apoptotic protein Bax, loss of mitochondrial membrane potential, release of cytochrome *c*, and activation of caspase-3 [17,18]. In addition, we demonstrated that GnRH-II antagonists couple to the GnRH-I receptor and have binding affinities to the GnRH-I receptor similar to those of the GnRH-I antagonist cetrorelix [18]. Furthermore, we showed that [D-Lys^6 ^]-GnRH-II is an agonist at the GnRH-I receptor, whereas GnRH-II antagonists are clear antagonists at the GnRH-I receptor [18].

About 50% to 64% of human breast cancers express GnRH-I receptors [19-22]. Because GnRH-II antagonists appear to be suitable drugs for efficacious and less-toxic endocrine therapy for endometrial and ovarian cancers, the question arises whether GnRH-II antagonist treatment could also be a new therapeutic option for breast cancers. Especially for triple-negative breast cancers, defined by the lack of both estrogen and progesterone receptors, as well as a lack of overexpression/amplification of the *HER2-neu *gene, the therapeutic options today are very limited.

In the present study, we analyzed whether a GnRH-II antagonist induces apoptosis in estrogen receptor/progesterone receptor-positive MCF-7 human breast cancer cells, which have a normal expression of *HER2-neu *and triple-negative MDA-MB-231 human breast cancer cells *in vitro *and *in vivo*. In addition, we ascertained whether knockdown of GnRH-I receptor expression affects GnRH-II antagonist-induced apoptosis, measured by loss of mitochondrial membrane potential and GnRH-II antagonist-induced apoptotic signaling, analyzed by activation of stress-activated MAPK p38 and caspase-3.

## Materials and methods

### Cell lines and culture conditions

The human breast cancer cell lines MCF-7 and MDA-MB-231 (triple-negative) were obtained from American Type Culture Collection (ATCC, Manassas, Virginia). To guarantee the identity of the cell lines over the years, the cells were expanded after purchase, and aliquots were stored in liquid nitrogen. Every half year, a new frozen stock was opened and expanded to carry out the experiments. The cells were cultured at 37°C in a humidified atmosphere of 5% CO_2 _in air, as previously described [8-10].

### Animals

Female athymic (nude) mice (CD1 nu/nu), 6 to 8 weeks old on arrival, were obtained from Charles River (Sulzfeld, Germany). The mice were housed in sterile cages in a temperature-controlled room with a 12-h light/12-h dark schedule and were fed autoclaved chow and water *ad libitum*. All experiments were done according to the German ethical guidelines and the German laws for protection of animals and were approved by the Lower Saxony State Office for Consumer Protection and Food Safety, Department for Animal Protection, Oldenburg, Germany.

### GnRH analogues

The GnRH-II antagonist [Ac-D2Nal^1^, D-4Cpa^2^, D-3Pal^3,6^, Leu^8^, D-Ala^10^]GnRH-II was developed by us and synthesized by Peptide Specialty Laboratories GmbH (Heidelberg, Germany) [17]. The GnRH-I agonist [D-Trp^6^]GnRH (triptorelin) was kindly provided by Ferring Pharmaceuticals (Copenhagen, Denmark).

### GnRH-I receptor knockdown

GnRH-I receptor knockdown was conducted as described previously [23]. A 43-bp fragment of the human GnRH-I receptor cDNA was cloned in antisense orientation (5'-CT AGA ACC ATG GAC TGT CCG ACT TTG CTG TTG CTT TTC AAA GC-3') into the *Nhe*I/*Sal*I sites of the eukaryotic expression vector, pIRES (Clontech, Palo Alto, California, USA), to produce the pGnRH-IR-antisense vector. Cells were grown to approximately 50% confluence on Nunc two-well chamber slides (immune cytochemistry) or in Nunc 100-mm dishes (immunoblotting). Transfections were done by using Superfect liposome reagents and following the manufacturer's instructions (Qiagen). After 12 h, transfected cells and nontransfected control cells were treated with the GnRH-I agonist triptorelin (100 n*M*) to induce GnRH-I receptor protein internalization. Twelve hours later, the medium was changed, and 20 ml PBS-BSA or an appropriate dilution of the GnRH-II antagonist [Ac-D2Nal^1^, D-4Cpa^2^, D-3Pal^3,6^, Leu^8^, D-Ala^10^]GnRH-II was added, to a final concentration of 10^-7 ^*M*. Every 12 h, fresh PBS-BSA or GnRH-II antagonist was added. After 24 h of incubation, the medium was changed.

Knockdown of the GnRH-I receptor protein was evaluated with immune cytochemistry by using a monoclonal mouse anti-human GnRH-I receptor antibody (clone A9E4; Research Diagnostics, Flanders, NJ, USA) and immunoblotting by using a polyclonal rabbit antihuman GnRH-I receptor antiserum (Peptide Research Laboratories, Heidelberg, Germany), as described previously [23].

### Mitochondrial membrane potential

For determination of GnRH-II antagonist-induced loss of mitochondrial membrane potential, cells with and without GnRH-I receptor knockdown were treated without or with the GnRH-II antagonist (10^-9 ^and 10^-7 ^*M*) for 72 h. After incubation, the cells were washed with PBS once, and the mitochondrial membrane potential was detected by using the JC-1 mitochondrial membrane potential detection kit, according the instructions of the manufacturer (Biotium, Hayward, CA, USA).

### Western blot analysis

GnRH-II antagonist-induced activation of stress-activated MAPK p38 and of caspase-3 was analyzed with Western blot.

Polyclonal rabbit anti-human phospho-p38 and polyclonal rabbit anti-human p38 antibodies were obtained from Cell Signaling (Frankfurt/Main, Germany). Polyclonal rabbit anti-human active caspase-3 antibody was purchased from BD Pharmingen (Heidelberg, Germany), and polyclonal anti human β-actin antibody was from Sigma Aldrich (Deisenhofen, Germany).

For determination of GnRH-II antagonist-induced p38 activity, cells with (wild-type; WT) and without GnRH-I-receptor expression (GnRH-I receptor knockdown, KD) were treated without or with the GnRH-II antagonist (10^-7 ^*M*) for 45 min. For determination of GnRH-II antagonist-induced caspase-3 activity, cells with (wild-type; WT) and without GnRH-I-receptor expression (GnRH-I-receptor knockdown; KD) were treated without or with the GnRH-II antagonist (10^-9 ^*M *and 10^-7 ^*M*) for 48 h.

After incubation, cells were detached immediately with 0.5 g trypsin (Biochrom, Berlin, Germany) and 5 mmol EDTA in 1 l PBS/BSA. The pellets were washed twice with PBS and resuspended with CelLytic buffer (Sigma) containing protease inhibitors (Sigma). Equal amounts of protein per sample (40 μg) were used and diluted to equal volumes with Laemmli buffer. The cell lysates were separated on SDS-PAGE (10%, ProSieve 50 Gel Solution; Cambrex, Verviers, Belgium) under reducing conditions and transferred to nitrocellulose membranes (Hybond-ECL; GE Healthcare Europe, Munich, Germany). The nitrocellulose membranes were blocked with 5% instant skimmed-milk powder, spray-dried (Naturaflor; Töpfer GmbH, Dietmannsried, Germany) in TBST (137 m*M *NaCl, 2.7 m*M *KCL, 0.1% Tween 20, 25 m*M *Tris/Cl, pH 7.4) for 1 h at RT, washed with TBST, and then incubated at 4°C overnight with the appropriate antibody in an 1:500 (anti β-Actin) or a 1:1,000 dilution in TBST, and then, after washings, incubated at RT with horseradish peroxidase-conjugated anti-mouse IgG or anti-rabbit IgG (GE Healthcare Europe) at an 1:10,000 dilution in TBST for 1 h. After washings, specifically bound antibody was detected by using the enhanced chemiluminescence kit (ECL; Millipore, Schwalbach, Germany). The bands were analyzed by using the Kodak 1D image system (Kodak, New Haven, CT, USA).

### *In vivo *studies

Tumors were initiated by subcutaneous injection of 1 × 10^7 ^cancer cells into the right flank of female athymic (nude) mice (CD1 nu/nu). After 2 (MDA-MB-231) or 3 (MCF-7) weeks, all animals had developed solid tumors of about 200 mm^3^, and treatment was initiated. The *in vivo *experiments were done as follows: vehicle solution (control 1), 25 nmol of GnRH-I agonist triptorelin (control 2), or 25 nmol of GnRH-II antagonist per mouse (five mice per group) was injected intraperitoneally. Treatment was repeated every 2 days. Tumor volumes were measured on days 7, 11, 16, and 21 of treatment (MCF-7) or on days 4, 8, 12, and 16 of treatment (MDA-MB-231). The mice were killed after 21 (MCF-7) or 16 (MDA-MB-231) days.

### Statistical analysis

All experiments were repeated 3 times with different passages of the respective cell lines. The data were tested for significant differences with one-way analysis of variance followed by the Student-Newman-Keuls test for comparison of individual groups, after a Bartlett test had shown that variances were homogeneous.

## Results

### Effects of GnRH-II antagonist treatment on induction of apoptosis in MCF-7 and triple-negative MDA-MB-231 human breast cancer cells *in vitro *

**Figure 1:**
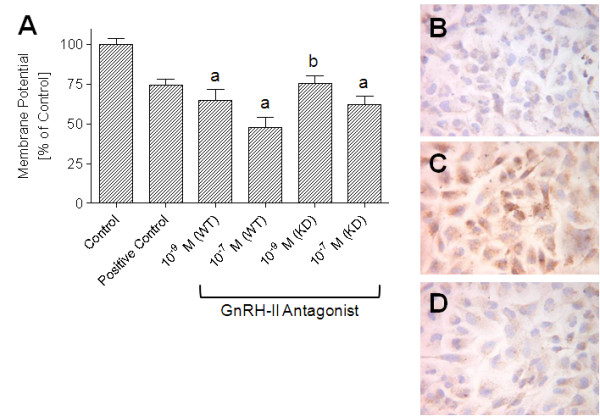
**Effects of GnRH-II antagonist treatment on induction of apoptosis in GnRH-I receptor-positive (wild-type; WT) and GnRH-I receptor-negative (GnRH-I receptor knockdown; KD) MCF-7 human breast cancer cells *in vitro***. **(a) **Percentage of mitochondrial membrane potential (ΔΨ) after 72 h of treatment of GnRH-I receptor-positive (wild-type; WT) and GnRH-I receptor-negative (GnRH-I receptor knockdown; KD) MCF-7 breast cancer cells without (control = 100%) or with cytotoxic agent doxorubicin (10^-9 ^*M*; positive control) or with GnRH-II antagonist [Ac-D2Nal^1^, D-4Cpa^2^, D-3Pal^3^, D-Lys^6^, D-Ala^10^]GnRH-II (10^-7 ^*M *and 10^-9 ^*M*). Columns represent mean ± SEM of data obtained from three independent experiments in three different passages of the cell line. **(a) ***P *< 0.001 versus control; **(b)***P *< 0.01 versus control. Experiments using MDA-MB-231 human breast cancer cells gave identical results. **(b-d) **Immune histochemical detection of GnRH-I receptor protein by using a monoclonal mouse anti-human GnRH-I receptor antibody. **(b) **Control performed by omission of the primary antibody. **(c) **Nontransfected cells. **(d) **Cells transfected with pGnRH-I-R antisense expression vector.

To analyze the effects of GnRH-II antagonist treatment on induction of apoptosis in estrogen receptor/progesterone receptor-positive MCF-7 human breast cancer cells, which have a normal expression of *HER1-neu *and triple-negative MDA-MB-231 human breast cancer cells, GnRH-II antagonist-induced loss of mitochondrial membrane potential (ΔΨ) was measured (Figure 1a). Treatment of MCF-7 and triple-negative MDA-MB-231 breast cancer cells with cytotoxic agent doxorubicin (10^-9 ^*M*; positive control) or with the GnRH-II antagonist (10^-9 ^and 10^-7 ^*M*) for 72 h resulted in a reduction of mitochondrial membrane potential. After treatment with 10^-7 ^*M *doxorubicin (DOX; positive control), mitochondrial membrane potential was decreased to 72.28 ± 3.98% of control (MCF-7; *P *< 0.01 versus control; Figure 1b) or to 76.49 ± 4.15% of control (MDA-MB-231; *P *< 0.01 versus control; not shown). Treatment with 10^-9 ^*M *GnRH-II antagonist resulted in a decrease of mitochondrial membrane potential to 64.51 ± 6.95% of control (MCF-7; *P *< 0.001; Figure 1a) or to 67.23 ± 4.32% of control (MDA-MB-231; *P *< 0.001 versus control; not shown). After treatment with 10^-7 ^*M *GnRH-II antagonist, the mitochondrial membrane potential was reduced to 47.94 ± 6.14% of control (MCF-7; *P *< 0.001 versus control; Figure 1a) or to 52.46 ± 3.37% of control (MDA-MB-231; *P *< 0.001 versus control; not shown).

### Effects of GnRH-II antagonist treatment on induction of apoptosis in MCF-7 and MDA-MB-231 human breast cancer cells *in vitro *after knockdown of GnRH-I receptor expression

To analyze whether the effects of the GnRH-II antagonist are mediated through the GnRH-I receptor, the expression of GnRH-I receptor protein was inhibited by using the antisense knockdown technique.

A 43-bp cDNA encoding a fragment of the human GnRH-I receptor was cloned in antisense orientation into the *Nhe*I/*Sal*I sites of the eukaryotic expression vector pIRES, to produce the pGnRH-I-R-antisense vector. Knockdown of the GnRH-I receptor protein was demonstrated with immune cytochemistry by using a monoclonal mouse anti-human GnRH-I receptor antibody (Figure 1b-d) and immunoblotting by using a polyclonal rabbit antihuman GnRH-I receptor antiserum (not shown; c.f. [23]). After internalization of the GnRH-I receptor induced by GnRH-I agonist triptorelin, a high density of novel synthesis of GnRH-I receptor protein, seen as GnRH-I receptor antigenicity, could be observed in nontransfected cells (Figure 1c), whereas transfected cells showed only slight GnRH-I receptor antigenicity (Figure 1d). As early as 1 day after knockdown of GnRH-I receptor expression, GnRH-I receptor protein antigenicity was significantly reduced in comparison with the control. After 2 days, no more GnRH-I receptor protein could be detected; however, 4 days later, a slight GnRH-I receptor protein antigenicity could be observed, indicating that the effectiveness of GnRH-I receptor knockdown begins to decrease after 6 days (not shown; c.f. [23]).

To analyze the effects of GnRH-II antagonist treatment on induction of apoptosis in GnRH-I receptor-negative (GnRH-I receptor knockdown) human breast cancer cells in comparison with the GnRH-I receptor-positive (wild-type) human breast cancer cells, GnRH-II antagonist-induced loss of mitochondrial membrane potential (ΔΨ) was measured (Figure 1a). After knockdown of GnRH-I receptor expression, GnRH-II antagonist-induced decrease of mitochondrial membrane potential was slightly reduced, as compared with the effect of GnRH-II antagonist treatment in nontransfected cells. Treatment of the GnRH-I receptor knockdown cell lines with 10^-9 ^*M *GnRH-II antagonist resulted in a decrease of mitochondrial membrane potential to 75.35 ± 4.64% of control (MCF-7; *P *< 0.01 versus control; not significant versus WT; Figure 1a) or to 78.26 ± 3.47% of control (MDA-MB-231; *P *< 0.01 versus control; not significant versus WT; not shown). After treatment of the GnRH-I receptor knockdown cell lines with 10^-7 ^*M *GnRH-II antagonist, mitochondrial membrane potential was reduced to 61.92 ± 5.27% of control (MCF-7; *P *< 0.001 versus control; not significant versus WT; Figure 1a) or to 64.93 ± 4.53% of control (MDA-MB-231; *P *< 0.01 versus control; not significant versus WT; not shown).

### Effects of GnRH-II antagonist treatment on apoptotic signaling in MCF-7 and MDA-MB-231 human breast cancer cells *in vitro *

**Figure 2:**
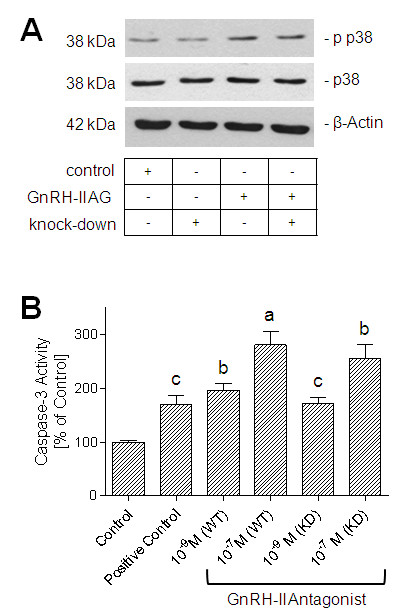
**Effects of GnRH-II antagonist treatment on apoptotic signaling in GnRH-I receptor-positive (wild-type; WT) and GnRH-I receptor-negative (GnRH-I receptor knockdown; KD) human breast cancer cells *in vitro***. **(a) **Amount of phosphorylated p38 after treatment without or with GnRH-II antagonist [Ac-D2Nal^1^, D-4Cpa^2^, D-3Pal^3,6^, Leu^8^, D-Ala^10^]GnRH-II (10^-7 ^*M*) in nontransfected MCF-7 breast cancer cells and in MCF-7 breast cancer cells after knockdown of GnRH-I receptor expression. These are representative data obtained from three independent experiments in three different passages of the cell line. Experiments using MDA-MB-231 human breast cancer cells gave identical results. **(b) **Percentage of caspase-3 activity after 48 h of treatment of GnRH-I receptor-positive (wild-type; WT) and GnRH-I receptor-negative (GnRH-I receptor knockdown; KD) MDA-MB-231 breast cancer cells without (control = 100%) or with cytotoxic agent doxorubicin (10^-9 ^*M*; positive control) or with GnRH-II antagonist [Ac-D2Nal^1^, D-4Cpa^2^, D-3Pal^3^, D-Lys^6^, D-Ala^10^]GnRH-II (10^-7 ^*M *and 10^-9 ^*M*). Columns represent mean ± SEM of data obtained from three independent experiments in three different passages of the cell line. **(a) ***P *< 0.001 versus control; **(b) ***P *< 0.01 versus control; **(c) ***P *< 0.05 versus control.

To analyze the effects of GnRH-II antagonist treatment on apoptotic signaling, GnRH-II antagonist-induced activation of stress activated MAPK p38 (Figure 2a) and caspase-3 (Figure 2b) in MCF-7 and MDA-MB-231 human breast cancer cells was measured.

Activation of p38 was considerably increased after GnRH-II antagonist treatment (Figure 2a, third band) as compared with the untreated control (Figure 2a, first band). The effects of the GnRH-II antagonist on induction of apoptotic cell death could further be confirmed by measurement of caspase-3 activity. Treatment of MDA-MB-231 human breast cancer cells with cytotoxic agent doxorubicin (10^-9 ^*M*; positive control) or with of the GnRH-II antagonist (10^-9 ^and 10^-7 ^*M*) for 48 h resulted in increased caspase-3 activity (Figure 2b). After treatment with 10^-9 ^*M *doxorubicin (positive control), caspase-3 activity was increased to 168.8 ± 17.54% of control (MDA-MB-231; *P *< 0.05; Figure 2b). Treatment with 10^-9 ^*M *GnRH-II antagonist resulted in an increase of caspase-3 activity to 195.8 ± 12.32% of control (MDA-MB-231; *P *< 0.01). After treatment with 10^-7 ^*M *GnRH-II antagonist, caspase-3 activity was increased to 279.8 ± 25.38% of control (MDA-MB-231; *P *< 0.001). MCF-7 human breast cancer cells are caspase-3 deficient. Therefore, no caspase-3 activation was detectable.

### Effects of GnRH-II antagonist treatment on apoptotic signaling in MCF-7 and MDA-MB-231 human breast cancer cells *in vitro *after knockdown of GnRH-I receptor expression

To analyze the effects of GnRH-II antagonist treatment on apoptotic signaling in GnRH-I receptor-negative (GnRH-I receptor knockdown) human breast cancer cells in comparison to the GnRH-I receptor-positive (wild-type) human breast cancer cells, we measured the GnRH-II antagonist-induced activation of stress activated MAPK p38 (Figure 2a) in GnRH-I receptor-negative (GnRH-I receptor knockdown) MCF-7 and MDA-MB-231 human breast cancer cells (Figure 2a). Effects of GnRH-I receptor knockdown on caspase-3 activation were analyzed in GnRH-I receptor-negative (GnRH-I receptor knockdown) MDA-MB-231 human breast cancer cells (Figure 2b). After knockdown of GnRH-I receptor expression, GnRH-II antagonist-induced activation of p38 was slightly reduced (Figure 2a, fourth band), as compared with the effect of GnRH-II antagonist treatment in nontransfected cells (Figure 2a, third band). The basal amount of phosphorylated p38 after knockdown of GnRH-I receptor expression alone (Figure 2a, second band) was only slightly decreased or nearly the same as compared with that in nontransfected cells without GnRH-II antagonist treatment (Figure 2a, first band). After knockdown of GnRH-I receptor expression, GnRH-II antagonist-induced caspase-3 activation was slightly reduced as compared with the effect of GnRH-II antagonist treatment in nontransfected cells. Treatment of the GnRH-I receptor knockdown cell lines with 10^-9 ^*M *GnRH-II antagonist resulted in an increase of caspase-3 activity to 170.6 ± 11.71% of control (MDA-MB-231; *P *< 0.05 versus control; not significant versus WT; Figure 2b). After treatment of the GnRH-I receptor knockdown cell lines with 10^-7 ^*M *GnRH-II antagonist, caspase-3 activity was increased to 254.5 ± 26.8% of control (MDA-MB-231; *P *< 0.01 versus control; not significant versus WT; Figure 2b).

### Effects of GnRH-II antagonist treatment on tumor growth *in vivo *

To show the proof-of-principle of an anti-tumor therapy using GnRH-II antagonists, nude mice bearing xenografted human breast tumors were treated subcutaneously with the GnRH-II antagonist [Ac-D2Nal^1^, D-4Cpa^2^, D-3Pal^3,6^, Leu^8^, D-Ala^10^]GnRH-II.

**Figure 3:**
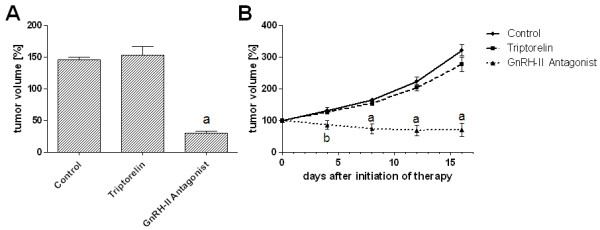
**Tumor volume of MCF-7 (a) and triple-negative MDA-MB-231 (b) human breast cancers xenografted into nude mice**. The mice were treated without (control 1), with 25 nmol of GnRH-I agonist Triptorelin (control 2) or with 25 nmol of GnRH-II antagonist [Ac-D2Nal^1^, D-4Cpa^2^, D-3Pal^3,6^, Leu^8^, D-Ala^10^]GnRH-II. Treatment was repeated every 2 days. Tumor volumes were measured on days 7, 11, 16, and 21 of treatment (MCF-7; **(a) **or on days 4, 8, 12, and 16 of treatment (MDA-MB-231; **(b)**). The mice were killed after 21 (MCF-7 **(a)**) or 16 (MDA-MB-231 **(b)**) days. Each experimental group consisted of five animals. Vertical bars represent SEM. **(a) ***P *< 0.001 versus control; **(b) ***P *< 0.05 versus control.

Female nude mice bearing MCF-7 (Figure 3a) or triple-negative MDA-MB-231 (Figure 3b) tumors s.c. were treated without (control 1) or with 25 nmol per injection of GnRH-I agonist triptorelin (control 2) or with 25 nmol per injection of the GnRH-II antagonist. The treatments were repeated every 2 days. The mice were killed after 16 (MDA-MB-231) or 21 (MCF-7) days of treatment. The increase of the tumor volume of the mice receiving therapy with GnRH-II antagonists was lower than that with the control animals. After 7 days of treatment, the differences on MCF-7 tumor growth (Figure 3a) became highly significant (*P *< 0.001) and remained highly significant (*P *< 0.001). The differences in MDA-MB-231 tumor growth (Figure 3b) became significant (*P *< 0.5) after 4 days of treatment. On day 8, the differences became highly significant (*P *< 0.001) and remained highly significant (*P *< 0.001). Side effects were not observed. To exclude that the antitumor effects of the GnRH-II antagonist are due to an interaction with the pituitary GnRH receptors, a second control group (control 2) was treated with 25 nmol per injection of the GnRH-I agonist triptorelin. The increase of the tumor volume of the mice receiving therapy with GnRH-I agonist triptorelin was nearly the same as that with the animals of control 1. No antitumor effects were observed with triptorelin.

## Discussion

Previous work showed that nanomolar concentrations of GnRH-II antagonists induce apoptotic cell death in human endometrial and ovarian cancer cells *in vitro *and *in vivo *[17]. In addition, we showed that apoptosis induced by GnRH-II antagonists is mediated through the intrinsic apoptotic pathway via stress-induced MAPKs p38- and JNK-induced activation of the proapoptotic protein Bax, loss of mitochondrial membrane potential, release of cytochrome *c*, and activation of caspase-3 [17,18]. Furthermore, we demonstrated that GnRH-II antagonists bind to the GnRH-I receptor and are clear antagonists at the GnRH-I receptor [18].

In the present study, we showed that treatment of estrogen receptor/progesterone receptor-positive MCF-7 and triple-negative MDA-MB-231 human breast cancer cells with the GnRH-II antagonist [Ac-D2Nal^1^, D-4Cpa^2^, D-3Pal^3,6^, Leu^8^, D-Ala^10^]GnRH-II resulted in apoptotic cell death via activation of stress-activated MAPK p38 and loss of mitochondrial membrane potential. In addition, GnRH-II antagonist-induced activation of caspase-3 could be observed in MDA-MB-231 human breast cancer cells. MCF-7 human breast cancer cells are caspase-3 deficient [24]. Therefore, no caspase-3 activation was detectable. Apoptotic signaling in MCF-7 cells differs from that in caspase-3-positive breast cancer cell lines, involving activation of different caspases. In MCF-7 cells, reduction of the mitochondrial membrane potential is caspase independent [25].

Triple-negative breast cancer refers to a specific subtype of breast cancer that is characterized by lack of expression of both estrogen and progesterone receptors as well as *HER2-neu*. Clinically, triple-negative breast cancer is more aggressive and less responsive to standard therapies and is associated with poorer overall patient prognosis [26,27]. To date, the therapeutic options are very limited, leaving chemotherapy the only possible therapy. Treatment with a GnRH-II antagonist could be a new option for the therapy of triple-negative breast cancer.

Recently we showed that GnRH-II antagonists bind to the GnRH-I receptor and are clear antagonists at the GnRH-I receptor [18]. However, after knockdown of GnRH-I receptor expression, GnRH-II antagonist-induced apoptosis and apoptotic signaling was only slightly reduced, indicating that the antitumor effects of GnRH-II antagonists are not exclusively mediated through the GnRH-I receptor. An additional pathway, such as the putative GnRH-II receptor, may be responsible for GnRH-II antagonist-induced apoptosis. The GnRH-I receptor-binding assays were carried out by using pituitary cells and fibroblasts [18]. Therefore, we cannot confirm a complete antagonism in tumor cells because the GnRH signal transduction is dependent on the cell context [28]. However, it cannot be ruled out that GnRH-II antagonists-induced apoptosis is mediated through the GnRH-I receptor. It would be very interesting to know whether GnRH-II antagonist-induced activation of apoptosis and apoptotic signaling would be abrogated after knockdown of expression of the putative additional receptor for GnRH-II antagonists. Different knockdown experiments, however, using GnRH-II receptor antisense fragments, resulted in apoptotic cell death (unpublished results). In addition, binding assays and functional assays for the putative GnRH-II receptor are not available. At present, therefore, this question cannot be answered.

We showed the proof-of-principle of an antitumor therapy by using the GnRH-II antagonist [Ac-D2Nal^1^, D-4Cpa^2^, D-3Pal^3,6^, Leu^8^, D-Ala^10^]GnRH-II *in vivo *in nude mice bearing subcutaneous xenografts of human breast cancers. Nude mice bearing MCF-7 human breast cancers or MDA-MB-231 triple-negative human breast cancers were treated with the GnRH-II antagonists [Ac-D2Nal^1^, D-4Cpa^2^, D-3Pal^3,6^, Leu^8^, D-Ala^10^]GnRH-II. The increase of the tumor volume of the mice receiving therapy with the GnRH-II antagonist was significantly lower than that with the control animals. Toxic side effects were not observed.

Our findings could be the basis for a further evaluation in clinical trials. GnRH-I agonists and GnRH-I antagonists have been widely used in the therapy for cancer [29-33] and endometriosis [34-36], as well as in reproductive medicine [37-41]. Their effects are mainly due to the downregulation of the hypothalamic-ovarian axis and the resulting medical castration. To exclude that the antitumor effects of the GnRH-II antagonist are mainly due to an interaction with the pituitary GnRH receptors and a subsequent reduction of ovarian estrogen production, control groups were treated with GnRH-I agonist triptorelin. Because we could not observe antitumor effects *in vivo *after downregulation of the hypothalamic-ovarian axis by using the GnRH-I agonist triptorelin, the downregulation of the hypothalamic-ovarian axis alone cannot be responsible for the antitumor effects of GnRH-II antagonists. In addition, triple-negative MDA-MB-231 breast cancer cells are insensitive to estrogen deprivation. The GnRH-II antagonist seems to affect the tumor cells directly by inducing apoptosis.

## Conclusions

We showed that the GnRH-II antagonist [Ac-D2Nal^1^, D-4Cpa^2^, D-3Pal^3,6^, Leu^8^, D-Ala^10^]GnRH-II induces apoptotic cell death in MCF-7 and triple-negative MDA-MB-231 human breast cancer cells *in vitro *and *in vivo*. Apoptosis induced by the GnRH-II antagonist is mediated through the intrinsic apoptotic pathway via the activation of stress-induced MAPK p38 and the loss of mitochondrial membrane potential. GnRH-II antagonist-induced activation of caspase-3 could be observed in MDA-MB-231 human breast cancer cells. In addition, we showed that knockdown of GnRH-I receptor expression only slightly inhibited GnRH-II antagonist-induced apoptosis and apoptotic signaling, indicating an additional pathway mediating the effects of GnRH-II antagonists.

Thus, the GnRH-II antagonist [Ac-D2Nal^1^, D-4Cpa^2^, D-3Pal^3,6^, Leu^8^, D-Ala^10^]GnRH-II seems to be a suitable drug for an efficacious and less-toxic endocrine therapy for breast cancers, including triple-negative breast cancers.

## Abbreviations

Ac-D2Nal: N-acetyl-d-2-naphthylalanine; BSA: bovine serum albumin; cDNA: complementary deoxyribonucleic acid; D-4Cpa: D-4-cyclopropylalanine; D-3Pal: D-3-(3-pyridyl)-alanine; D-Ala: D-alanine; Δψ: mitochondrial membrane potential; DOX: doxorubicin; EDTA: ethylenediaminetetraacetic acid; GnRH: gonadotropin-releasing hormone; HER2-neu: human epidermal growth factor receptor 2; IgG: immunoglobulin G; JNK: c-Jun N-terminal kinase; KD: knockdown; Leu: leucine; MAPK: mitogen-activated protein kinase; PBS: phosphate-buffered saline; pIRES: plasmid containing the encephalomyocarditis virus internal ribosome entry site flanked by two multiple cloning sites; RT: room temperature; SDS-PAGE: sodium dodecylsulfate-polyacrylamide gel electrophoresis; TBST: tris-buffered saline Tween-20; WT: wild type.

## Competing interests

The authors declare that they have no competing interests.

## Authors' contributions

CG designed the experiments, performed data analysis and statistics, and drafted the manuscript. CF, SF, and NN participated in the design of the experiments and carried out the *in vitro *and *in vivo *experiments. ARG participated in the study design and helped with data analysis. GE helped with data analysis and statistics and helped to draft the manuscript. All authors read and approved the final manuscript.
